# Characteristics of pulsed runoff-erosion events under typical rainstorms in a small watershed on the Loess Plateau of China

**DOI:** 10.1038/s41598-018-22045-x

**Published:** 2018-02-27

**Authors:** Lei Wu, Jun Jiang, Gou-xia Li, Xiao-yi Ma

**Affiliations:** 1 Key Laboratory of Agricultural Soil and Water Engineering in Arid and Semiarid Areas, Ministry of Education, Northwest A&F University, Yangling, 712100 P.R. China; 2 Ansai Comprehensive Experimental Station of Soil and Water Conservation, Chinese Ecosystem Research Network, Northwest A&F University, Yangling, 712100 P.R. China; 3 Department of Civil and Environmental Engineering, University of California, Berkeley, California 94720 USA; 4 College of Water Resources and Architectural Engineering, Northwest A&F University, Yangling, 712100 P.R. China; 5 State Key Laboratory of Soil Erosion and Dryland Farming on the Loess Plateau, Northwest A&F University, Yangling, 712100 P.R. China

## Abstract

The pulsed events of rainstorm erosion on the Loess Plateau are well-known, but little information is available concerning the characteristics of superficial soil erosion processes caused by heavy rainstorms at the watershed scale. This study statistically evaluated characteristics of pulsed runoff-erosion events based on 17 observed rainstorms from 1997–2010 in a small loess watershed on the Loess Plateau of China. Results show that: 1) Rainfall is the fundamental driving force of soil erosion on hillslopes, but the correlations of rainfall-runoff and rainfall-sediment in different rainstorms are often scattered due to infiltration-excess runoff and soil conservation measures. 2) Relationships between runoff and sediment for each rainstorm event can be regressed by linear, power, logarithmic and exponential functions. Cluster Analysis is helpful in classifying runoff-erosion events and formulating soil conservation strategies for rainstorm erosion. 3) Response characteristics of sediment yield are different in different levels of pulsed runoff-erosion events. Affected by rainfall intensity and duration, large changes may occur in the interactions between flow and sediment for different flood events. Results provide new insights into runoff-erosion processes and will assist soil conservation planning in the loess hilly region.

## Introduction

Soil erosion by water is one of the most widespread and major ecological environmental problems worldwide, which results in reduced agricultural productivity, increased water pollution, and unsustainable development^1–5^. Soil erosion is a complex physical process, the fundamental explanations are the comprehensive interaction between precipitation and the surface of watersheds^6,7^. In a mathematical sense, it is a highly non-linear mapping relationship from the watershed surface and rainfall conditions to runoff and sediment transport^8,9^. Generally, the main factors affecting rainfall erosion and sediment yield include: rainfall intensity, rainfall duration, rainfall spatio-temporal characteristics, soil properties, geological conditions, vegetation, land use and antecedent soil wetting condition^10,11^. Rainfall is one of the most important active agents of soil erosion, due to its potential to breakdown aggregates, detach soil particles, and produce runoff^12–14^. At present, many scholars have conducted experimental and modelling studies in runoff and sediment yield characteristics under the conditions of different soil types, rainfall intensity, slope gradient, land use types, vegetation coverage, and water conservation measures^15^. However, most studies mainly explored runoff and sediment loss on runoff plots^16,17^. On the other hand, other studies focused on rainfall, runoff and sediment relationships at basin scales. They mainly analysed relationships between rainfall runoff and sediment yield on annual time scales^18^. Besides, some scholars have also constructed different rainstorm runoff-sediment models, but they did not reveal the pulsed runoff-sediment relationship of the typical loess hilly watershed, due to local characteristics^19,20^.

Generally, studies on runoff and sediment change on the Loess Plateau are based on model simulation or measured data analysis. The model simulation method has better prospects for causal analysis of runoff and sediment changes through flexible parameter adjustment^21,22^. However, the model can only be used to abstract and generalize natural phenomena, and cannot fully reveal the occurrence and evolution mechanism of natural phenomena^23^. Therefore, it is still an effective method to study the variation of runoff and sediment using measured data. Studies have shown that research on runoff and sediment change using measured data has the following characteristics^24^: (i) The spatial scales are mainly concentrated on runoff plots and slope surfaces when investigating the influence of rainfall intensity and vegetation on rainfall-runoff and sediment yield. These small-scales can not sufficiently reflect the regional variation rules of water and sediment for large spatial scales^25,26^. (ii) The impact of annual rainfall on runoff and sediment has been considered to be a key priority when the spatial scale expands to larger areas, but the influencing effects of rainfall intensity during one precipitation on runoff and sediment yield in large spatial scales will be weakened^27,28^. (iii) Statistical analysis is an important method to study rainfall-runoff interactions^29^. Cluster Analysis is an unsupervised learning process to find one set of similar elements in a data set, it is of great significance to policy decisions on soil and water conservation. Therefore, it is necessary to study correlations between runoff and sediment yield under individual monitoring rainstorm events in a typical small watershed of the Loess Plateau.

The Loess Plateau is located in an arid and semi-arid area. Annual rainfall ranges between 200–600 mm. Soil erosion on the Loess Plateau is very serious. Erosion is mainly caused by several heavy rainstorms during 6–9 months, and the amount of erosion by heavy rains accounts for >60% of total annual erosion^30^. So storm runoff erosion is the main pathway of sediment yield and the main power of sediment transport for other erosion processes^31^. Due to the high intensity and short duration of the few heavy rains, rainstorms on loess slopes rapidly form infiltration-excess runoff and cause very high runoff rates^32^. Because loess soil is highly erodible, a large amount of infiltration-excess runoff also results in excessive erosion, which makes this region one of the most eroded areas in the world^33^. Therefore, according to the erosion characteristics of the Loess Plateau, it has more significance for the establishment of soil erosion models and soil conservation by predicting runoff and sediment yield relationships of individual rainstorm events than studies on annual rainfall-erosion relationships^34^. Only clearly knowing the characteristics of erosion and sediment yield under typical pulsed runoff-erosion events will allow effective scientific management of soil and water conservation at watershed scales.

The main aims of this paper are to: (1) investigate characteristics of pulsed runoff-erosion events in a loess hilly-gully watershed, and (2) evaluate the influencing mechanisms of relationships between rainstorm and sediment yield. Results may provide the theoretical basis for the construction of rainstorm runoff and sediment yield models and for decision-making in watershed management.

## Study area

**Figure 1:**
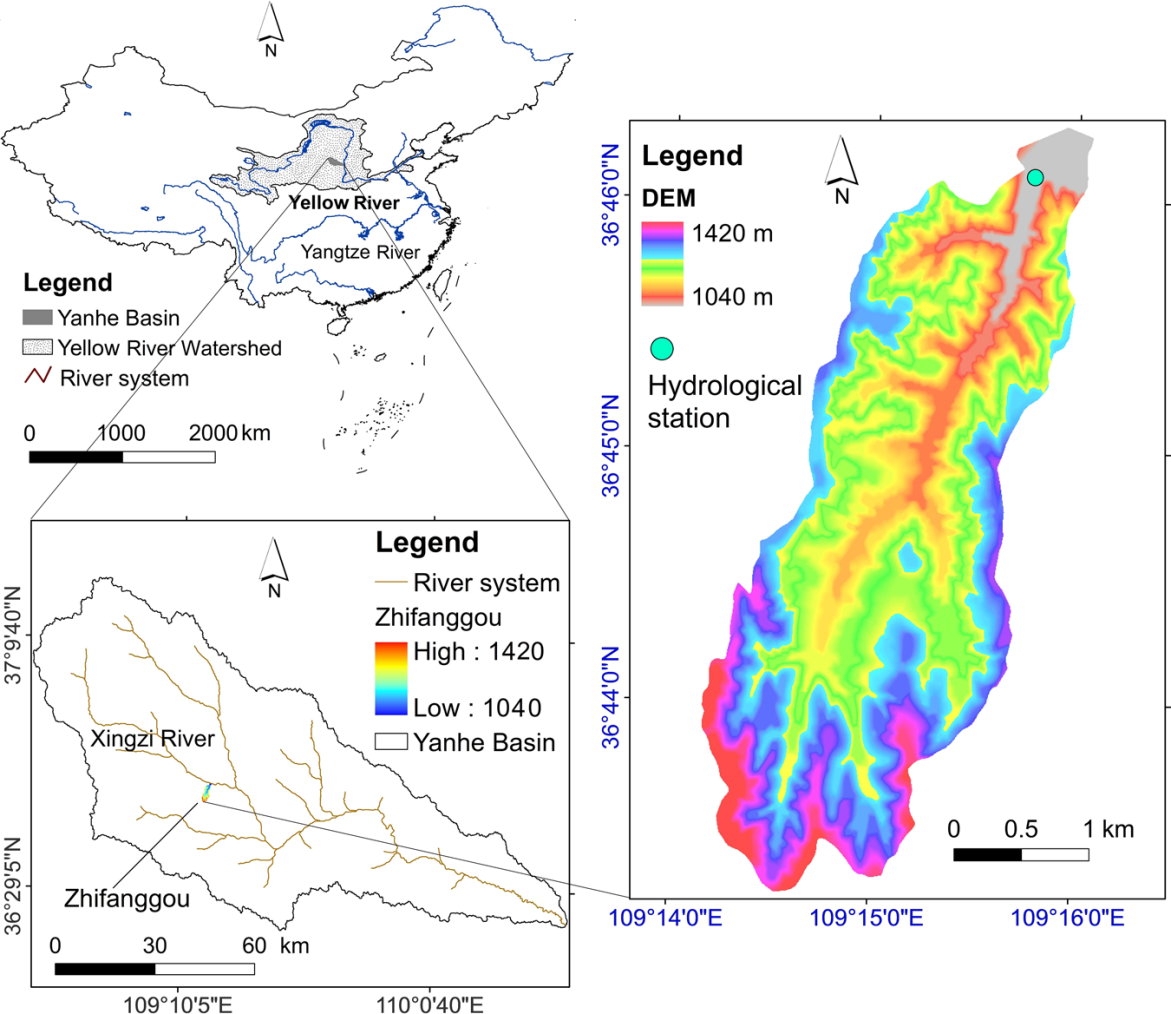
The relative location of Zhifanggou Watershed and the Yanhe River basin, the Digital Elevation Model of Zhifanggou Watershed (Fig. 1 is generated by ArcGIS 10.2, Yanhe River is a primary tributary of the Yellow River. The Yanhe River boundary and river system are extracted by hydrological analysis tools, Zhifanggou Watershed is overlayed by the DEM map and the hillshade map. ArcGIS website: http://www.esrichina.com.cn/softwareproduct/ArcGIS/).

Zhifanggou is a first order tributary of the Xingzi River, which lies in the middle and upper reaches of the Yanhe River in Ansai County, Shaanxi Province. Zhifanggou Watershed (109°13′46″-109°16′03″E and 36°42′42″-36°46′28″N) belongs to No. 2 sub-region of the hilly and gully area in the Loess Plateau (Fig. 1). The watershed area is 8.27 km^2^ and the elevation is between 1040–1425 m above sea level. The relative altitude difference of the upstream and downstream river bed is 210 m and the mean river slope is 37‰. Most of the height differences between the hill top and valley are 150–200 m. Soil erosion is dominated by water erosion and gravity erosion. Sheet erosion, rills and ephemeral gullies are the main erosion forms on the hilly slopes, and gully erosion and gravity erosion are the main erosion forms on the gully slopes^35^.

The watershed belongs to a transition zone between semi-humid and semi-arid in the warm temperate climate, the mean annual temperature is 8.8 °C and mean annual precipitation is 482.7 mm. Rainfall mainly falls as rainstorms and is concentrated in June-September, which accounts for 73.6% of the total annual rainfall amount.

**Figure 2:**
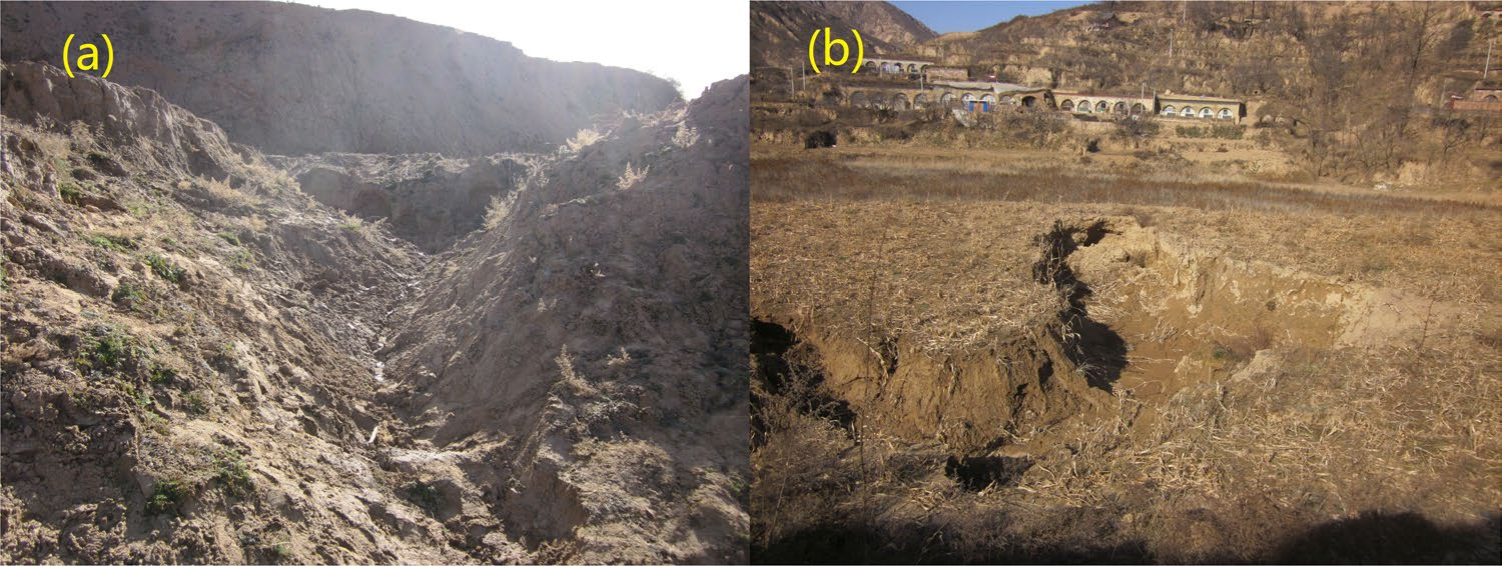
Gully erosion of slope surface (**a**) and flat arable land (**b**) in Ansai County of the Yanhe River Basin in 2013 (The pictures were taken by Lei Wu in Ansai County in November 2013).

The main soil type in the watershed is loess according to the Chinese soil classification system in 1995. The loess area accounts for 65.5% of the total soil area, following by 25.1% of Red Soil and Two Coloured Soils. Soil mechanical composition includes clay (<0.002 mm, 53.9–74.8%, silt (0.002~0.05 mm, 16–26%), and sand (0.05~2 mm, 5.88–31.8%)^36^. Soil has a uniform texture, with low organic matter content (6.82–34.6 g/kg) and loose structure, and can be easily dispersed and transported (Fig. 2). Before watershed governance, the watershed had sparse vegetation, high planting rates and serious soil erosion^37^. The mean sediment yield modulus for many years was ≤14 000 t/(km^2^•a). Since integrated basin management started in 1997, the watershed was vegetated by afforestation and grass planting^38^.

## Materials and watershed monitoring

Seventeen typical pulsed rainstorms were observed in Zhifanggou Watershed from 1997–2010, to study rainfall, runoff and sediment relationships. The 17 rainstorms have their own local characteristics of short duration and high intensity, resulting in different interactions between runoff and sediment yield. In the 17 rainstorms, rainfall ranged from 13.4 mm on 4 May 1997 to 56.4 mm on 20 May 1998, with rainfall durations of 150 minutes and 330 minutes, respectively. In August 2010, two short high intensity rainfall events occurred, 50 and 60 minutes rainfall duration on 11 August 2010 and 18 August 2010 with 55.6 and 40.5 mm rainfall, respectively.

The monitoring indexes of soil and water conservation in a small watershed includes rainfall, water level, flow rate and flood sediment (suspended sediment). In this study, two methods, including rain gauge and automatic rain recorder, were used to measure rainfall amount in the watershed. Three rainfall stations were established in the central, upper and lower reaches of the watershed, respectively. The simultaneous measurement of rain gauge and automatic rain recorder were checked with each other and the mean value taken as the watershed rainfall. Water-level observation is one of the basic measurements at the runoff station, and is the basis of flow estimation. Water level was measured by water gauge, and the flow rate from the watershed outlet was calculated using a flume weir flow formula^39^. The basic requirements of water-level observation at the basin outlet are: the daily water level was measured at 0800 and 2000 (local time) during normal water-level periods, and the flood level was measured by points of flood rising, falling and water-level changing at different short intervals^40^. Observation accuracy was ±1 cm. Sediment was analysed by collecting runoff samples. The observation of suspended sediment is carried out by artificial observation method. In the process of runoff yield, the turbid water samples were collected artificially by a sampling bottle at a certain time interval, the sediment content was measured by drying in the laboratory, the sediment yield of the whole basin was then calculated^41^. The volume of the sample bottle is 1000 mL. Sampling times were 3, 6, 12 and 30 minutes after runoff yield during one rainstorm.

Regression analysis is an important method to study relationships between runoff and sediment yield in small watersheds^42^. In this study, the statistical analysis software (Excel & SPSS) was used to analyse the runoff and sediment relationships of typical rainstorm events^43^. Besides, Cluster Analysis (MATLAB Procedure) is an important approach of unsupervised learning^44^. As a data analysis tool, its importance has been widely recognized in various fields. A Cluster Analysis is performed in order to find the ‘natural grouping’ of data sets, which is also called a ‘cluster’. Generally, a cluster is a collection of similar elements. Similarity coefficient and distance are two important parameters of Cluster Analysis. The similarity coefficient is indicative of the similarity degree between different sample variables, the range is 0–1. When its value is close to 1, the similarity degree between samples is strong. If the value is 0, there is no correlation between samples. Distance represents the geometric distance between different points, and the distance is related to the attributes of the sample points. The basic idea of Cluster Analysis is: first “*n*” samples are classified as one class separately, and the most similar of them is classified as a new class, at this point, the number of categories becomes “*n* − 1”. Then, the similarity between the new class and other “*n* − 2” classes is calculated, and the nearest approximation is classified as another new class, the total class number becomes “*n* − 2”. By analogy, the process continues until all variables are classified as one category. This clustering process can be expressed by the cluster map. This study used 153 data sets of 17 sets and 9 categories as the sample data (Table 1). Firstly, these data are standardized to eliminate the dimensional differences, so that the indexes are comparable. Secondly, the distance or similarity coefficient between different variables is calculated. Then, the appropriate classification methods are selected and used to progressively cluster samples.

**Table 1:** Rainfall runoff and sediment yield parameters and Cluster Analysis results during 17 event-based rainstorms.

Date	Cluster results	Rainfall (mm)	Runoff yield time (s)	Mean flow (m^3^/s)	Peak flow (m^3^/s)	Total flow (m^3^)	Runoff depth (mm)	Runoff coefficient	Mean sediment concentration (g/cm^3^)	Suspended sediment discharge (t)	Sediment yield modulus (t/km^2^)
1997–4–5	1	13.4	9000	0.0073	0.0111	80.51	0.0097	0.0007	0.480	38.65	4.67
1997-6-5	1	16.4	7560	0.0067	0.0094	48.99	0.0059	0.0004	0.379	18.56	2.24
1997-28-7	2	14.2	16200	0.0096	0.0151	141.37	0.0171	0.0012	0.392	55.40	6.70
1998-21-5	2	56.4	19800	0.0106	0.0174	310.95	0.0376	0.0007	0.070	21.71	2.63
1998-23-6	5	24.6	16200	2.6034	12.242	16051.59	1.9409	0.0927	0.196	3140.76	379.78
1998-12-7	2	20.9	23400	0.0086	0.014	228.59	0.0276	0.0013	0.104	23.73	2.87
1998-20-8	2	30.6	16200	0.2567	0.6208	2826.8	0.3418	0.0112	0.049	139.70	16.89
2000-7-7	5	34.8	15000	2.3986	7.4811	17602.54	2.1285	0.0612	0.260	4570.58	552.67
2000-14-7	6	41.0	17640	4.5047	13.2777	44026.09	5.3236	0.1298	0.248	10920.67	1320.52
2000-26-7	8	44.5	14400	8.9263	17.2954	64942.41	7.8528	0.1765	0.256	16618.76	2009.52
2000-11-8	7	16.3	14400	2.4128	5.2329	36247.42	4.3830	0.2697	0.204	7399.33	894.72
2001-26-7	5	46.7	12600	1.3001	4.7276	17283.95	2.0900	0.0448	0.102	1759.51	212.76
2001-16-8	4	31.8	13680	3.5062	7.5814	26541.13	3.2093	0.1009	0.124	3296.41	398.60
2008-28-6	5	18.6	16200	1.3211	2.0215	16774.84	2.0284	0.1091	0.083	1394.09	168.57
2009-7-7	1	34.5	5400	0.0069	0.0094	19.11	0.0023	0.0001	0.014	0.26	0.03
2010-11-8	3	55.6	3000	4.0090	6.0731	12852.17	1.5541	0.0333	0.076	970.77	117.38
2010-18-8	3	40.5	3600	2.2924	3.4577	8032.22	0.9712	0.0208	0.023	185.95	22.48

Note: The similarity coefficient value is 0.8267 by programming calculation of Cluster Analysis.

## Results and Discussion

### Relationships between runoff and sediment concentrations for each rainstorm event

**Figure 3:**
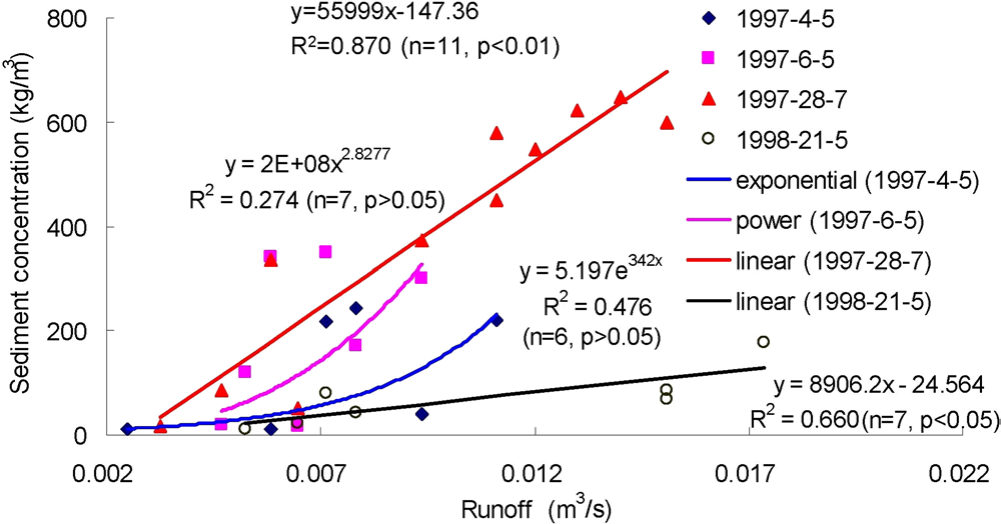
Relationships between sediment concentration (0–800 kg/m^3^) and runoff (0.002–0.022 m^3^/s) under different rainstorm events.

**Figure 4:**
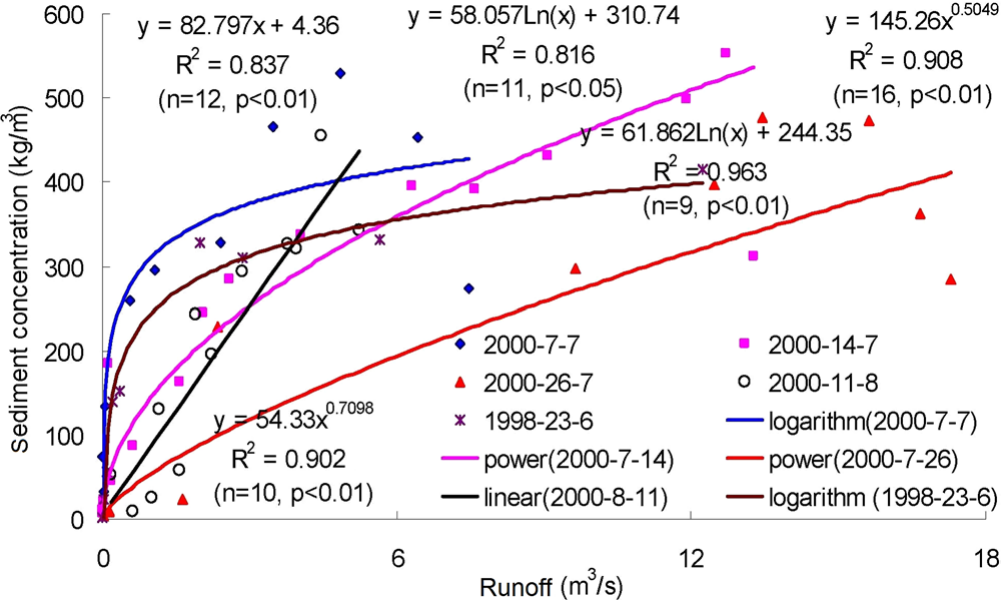
Relationships between sediment concentration (0–600 kg/m^3^) and runoff (0–18 m^3^/s) under different rainstorm events.

**Figure 5:**
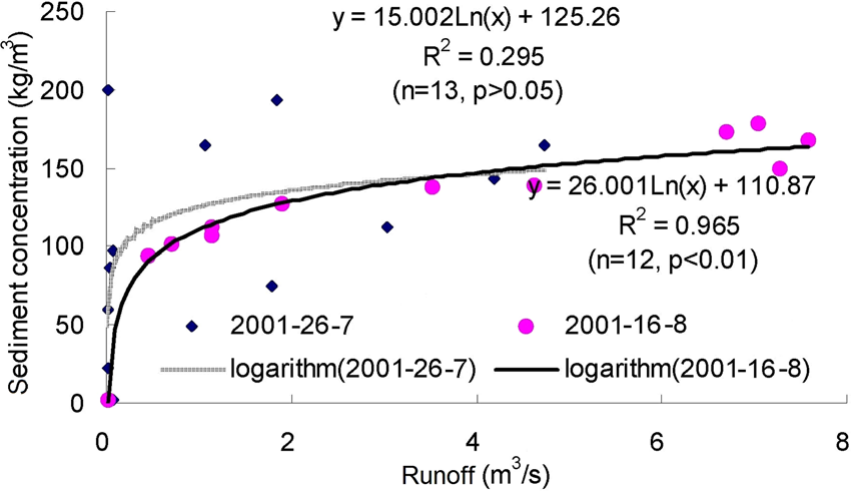
Relationships between sediment concentration (0–250 kg/m^3^) and runoff (0–8 m^3^/s) under different rainstorm events.

**Figure 6:**
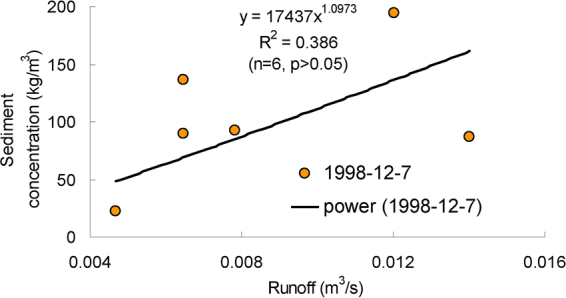
Relationships between sediment concentration (0–200 kg/m^3^) and runoff (0.004–0.016 m^3^/s) under different rainstorm events.

**Figure 7:**
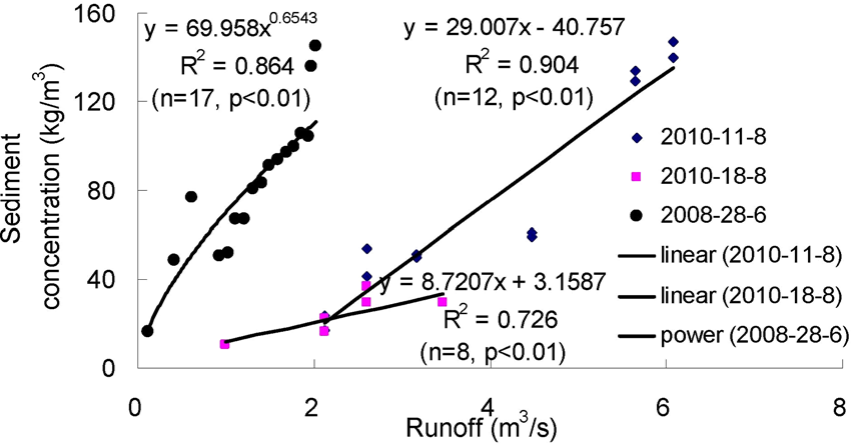
Relationships between sediment concentration (0–160 kg/m^3^) and runoff (0–7 m^3^/s) under different rainstorm events.

**Figure 8:**
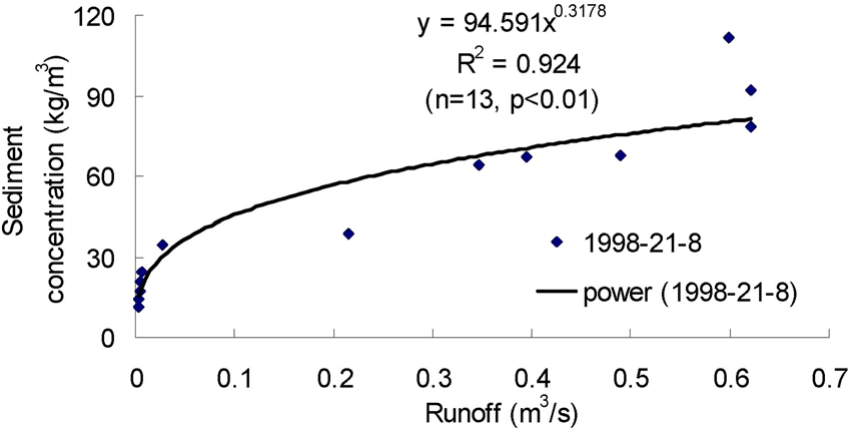
Relationships between sediment concentration (0–120 kg/m^3^) and runoff (0–0.7 m^3^/s) under different rainstorm events.

**Figure 9:**
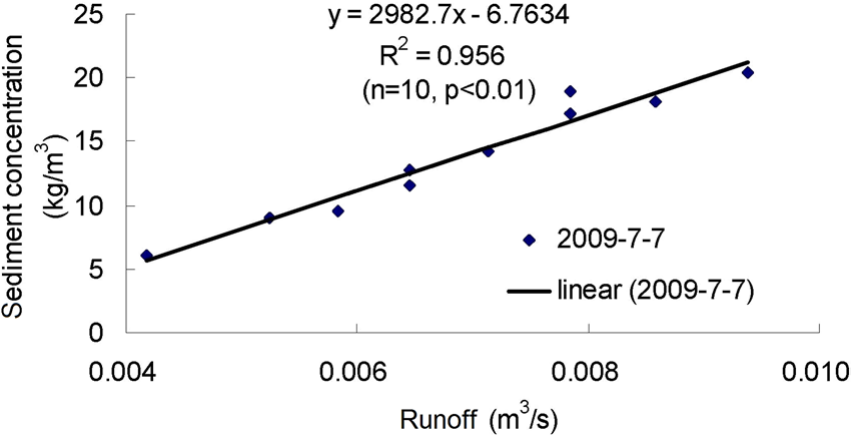
Relationships between sediment concentration (0–25 kg/m^3^) and runoff (0–0.01 m^3^/s) under different rainstorm events.

Figures 3–9 show the regression relationships between sediment concentrations and runoff amount under different rainstorm events. Correlations between runoff and sediment yield are diverse under different rainstorm events. Generally, sediment concentrations increase with increased flow rate, but when sediment concentration reaches a certain value, the change of sediment concentration is very small, even if the flow continuously increases. The value eventually tends to stabilize. It also shows that the influence of flow rate on sediment concentration has different effects during different rainfall erosion events. Therefore, the corresponding curve fitting can also be changed in different rainstorm events^45^. In all rainstorm erosion events, there is not always a strong correlation between runoff and sediment yield. Statistical analysis of 17 rainstorm events found that the regression relationships of 4 May 1997, 6 May 1997, 12 July 1998 and 26 July 2001 were weak, R^2^ (coefficient of determination) were all <0.5 and p values were all >0.05. The values of R^2^ for the rainstorm events on 18 August 2010 and 21 May 1998 are both <0.8, but there were three rainstorm events when R^2^ values were all >0.95 and p values were all <0.01 (23 June 1998, 26 August 2001 and 7 July 2000).

In terms of the strongest correlations, 17 rainstorm erosion events include linear, power, logarithmic and exponential functions. The logarithmic function is the upper convex curve, which is characterized by sediment yield increasing sharply with increased runoff (Figs 3–9). Representative rainstorm events are 7 July 2000, 23 June 1998, and 16 August 2001. The erosion amount of the three rainstorms roughly lies between 3000–5000 t. The power function can be divided into the upper convex or lower convex type according to the size of the index. Representative rainfall events are 26 July 2000, 14 July 2000, 28 June 2008 and 21 August 1998. The sediment yield rate with increased runoff is lower than the logarithmic function during the initial runoff yield period. The sediment yield rate of the linear regression relation is between power function and logarithmic function. Representative rainfall events are 28 July 1997, 11 August 2000, 11 August 2010 and 7 July 2009. The regression relationships on 6 May 1997 and 26 July 2001 were the weakest. The 26 July 2001 was a heavy rainfall erosion event. The 6 May 1997 was a prolonged rainfall event. Because the project of returning farmland to forest in 1997 was in the primary stage, the relationship between sediment yield and runoff was unstable. In 2009, with the effectiveness of soil and water conservation measures, the sediment yield of small rainstorm events was linearly correlated with runoff. A representative rainfall event was 7 July 2009. These results also validated that the surface conditions on the loess slope significantly affected runoff and sediment yield processes^46^. The above analysis is of great significance for further summarizing relationships between runoff and sediment yield, and understanding the variation of sediment yield associated with different rainstorm events.

The various regression associations between runoff and sediment yield are related to the characteristics of both rainstorms and soil erosion on the Loess Plateau. Generally, the storms in the north of Shaanxi Province mostly appear at a single station and single time, indicating that the most storms in this region are usually local ones with short duration^47^. Both rill and shallow gully erosion are very serious on the Loess Plateau, and gully erosion is also very active. The sediment yield of gully erosion accounts for >70% of total sediment yield in the watershed. Although the rills and shallow gullies are small, they are numerous, and dense networks cover all loess slopes. During periods of infiltration-excess rainstorms, overland flow always flows along the direction of minimum resistance, because of the spatial difference of soil erosion resistance, and forms a small stream of relatively concentrated runoff on the slope, and then develops into different erosion types. The rill, shallow gully, dissected valley and gully in Zhifanggou Watershed form a dense network, and play very important roles in the formation of pulsed runoff-erosion events. The weak erosion resistance of loess soil is the fundamental reason for the severe erosion. Under the action of water flow, the stability of sediment particles is determined by the size of sediment particles and the cohesive forces between them^48^. For coarse-grained sediment, size plays a major role in stability, and the larger the particle size, the greater the starting velocity. When the particle size is less than a certain value, fine particles are also difficult to move, and the smaller the particle size, the larger the starting velocity. In general, fine and silt sand particles are most easy to erode, and the content of silt sand particle is the highest and more than >50% of the loess soil, but the starting velocity of clay soil particles is slightly larger.

For slopes without rill or shallow gully erosion, the final destination of filling water in the early excess infiltration rainstorm is infiltration. After overflow develops on slopes, sheet overflow will finally enter the channel. Therefore, in this case, the confluence speed is small, the flood duration is long, and the ratios of flood peak and discharge amount are reduced^49^. On the slope with rill or shallow gully erosion, the filling water in the early period of infiltration-excess rainstorm appears as a ‘dam break effect’ after appearance of the rill or gully erosion during the rainstorm. It suddenly releases by way of ‘installment time deposit’, and rapidly changes into centralized and strong channel flow^50^. This phenomenon reduces infiltration of rainwater into soil, increases the surface runoff coefficient, and makes the flood show features of “high peak and small volume, less runoff and much sand, peak with rising and dropping steeply, sudden coming and rapid going away”^51^. The characteristics of rainstorm erosion have important influences on relationships between runoff and sediment yield.

### Relationships of rainfall-runoff and rainfall-sediment during different rainstorms

Rainstorms on the Loess Plateau have highly variable spatio-temporal characteristics^52^. Heavy rainfall is concentrated in a few hours or even minutes. There are also major differences for the same rainstorm within several kilometres; it has great locality. The typical spatio-temporal characteristics of rainstorms determine the dominant position of infiltration-excess runoff on the Loess Plateau, and the flood process showed a precipitous fall with a sharp thin peak. Runoff generation was mainly driven by precipitation characteristics and the initial catchment saturation^53^. Because of the particularity of hydrological processes and the implementation of soil conservation measures on the Loess Plateau, the correlations of rainfall-runoff (*y* = 451.16e^0.0598×^, R^2^ = 0.101, *n* = 17, *p* > 0.05) and rainfall-sediment (*y* = 36509x − 4E + 06, R^2^ = 0.114, *n* = 17, *p* > 0.05) are often both scattered and very weak (Table 1), although the correlation of rainfall-sediment (mm, kg) is stronger than rainfall-runoff (mm, m^3^). The results are consistent with previous studies that the annual sediment yield of the Xiliugou Basin in the upper Yellow River showed a significant downward trend from 1960–2013, whereas no significant trend was detected in annual precipitation^54^. Furthermore, high vegetation cover and well-developed biological soil crust were the important factors in reducing runoff and erosion^55,56^. The results accord with Zhao *et al*. (2014), who showed pastures and crops were effective in decreasing runoff and erosion^57^. The relation of rainstorm and runoff can be expressed as the relation between total rainstorm amount and total runoff amount, or the relation between the spatio-temporal process of the rainstorm and the corresponding runoff yield.

The loess hilly region is a typical infiltration-excess runoff area. The main factors affecting runoff and sediment yield are rainfall intensity, rainfall duration and surface conditions (slope, gully density and antecedent moisture condition). Among them, the surface condition is a more deterministic factor, while rainfall is a stochastic factor^58^. Rainfall intensity in the loess hilly region plays a decisive role in runoff yield amount, while antecedent soil water content takes second place. Each stage of soil loss by unit runoff for a given mean rainfall intensity was significantly different among rainstorm patterns^59^. Besides, because the climate of the Loess Plateau is arid and semi-arid, the thickness of loess soil is often 50–80 m and the thickest is 150–180 m, the water storage capacity of the whole vadose zone is very large, low intensity rainfall often does not produce runoff, only intense rainstorms produce surface runoff. Due to the uneven spatio-temporal distribution of rainstorms, runoff yield from catchments is local, and extensive simultaneous runoff is extremely rare. Therefore, the flood process is often sudden rise and sudden fall, and the peak volume is small. Under the same conditions, if rainfall is large, runoff is generally large, and runoff erosion capacity and sediment transport capacity are also large. The more uneven the distribution of rainfall in time and space, the more concentrated the sediment yield in different periods, the greater the amount of runoff and sediment yield in the same rainfall event^60^.

### Relationships between total runoff and suspended sediment transport during different rainstorms

Soil erosion models are an important means of soil loss prediction and water conservation assessment^61^. The relationship between runoff and sediment is the basis of soil erosion models^62^. Most of the Loess Plateau is covered by large amounts of loess soil, it has characteristics of uniform texture, loose and porous structure, vertical joint development and high water permeability. The soil is erodible.

**Figure 10:**
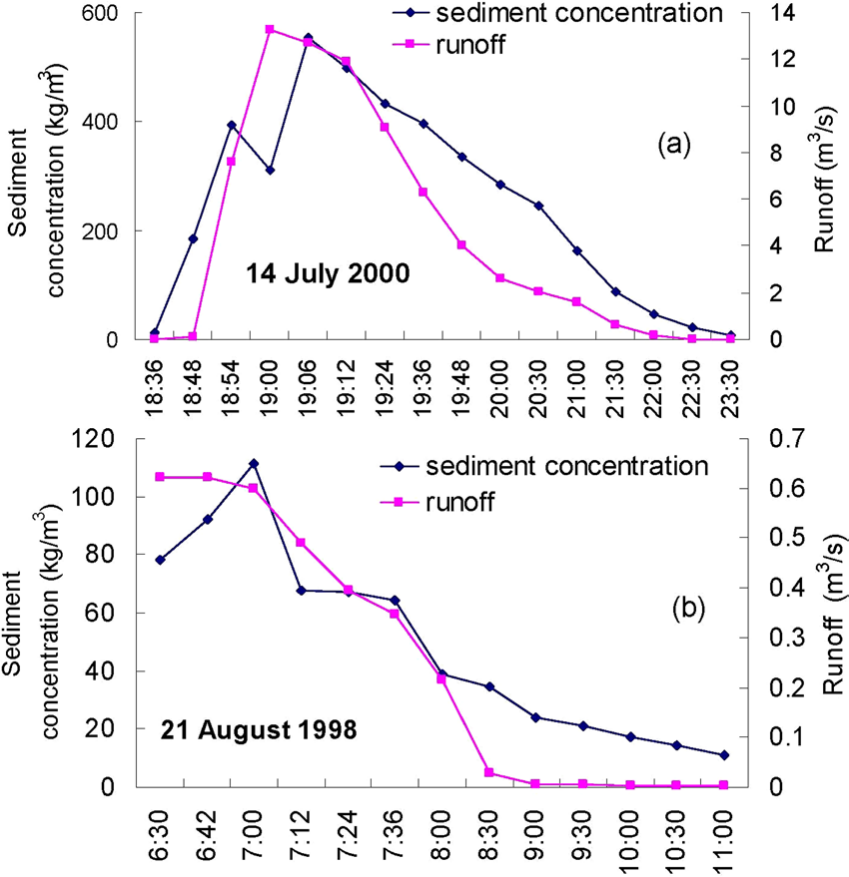
Flow and sediment hydrograph on (**a**) 14 July 2000 and (**b**) 21 August 1998.

The unit hydrograph is an important method to analyse relationships between runoff and sediment yield of event-based rainstorms. Figure 10 shows two hourly flow-sediment hydrographs of Zhifanggou Watershed under typical rainstorm events. Figure 10(a) indicates that sediment concentrations increased along with increased flow rate during the water rising period, and the water flow fades away quickly and sediment concentration decays slowly. The results agree well with Cao *et al*. (2015) that the initial soil losses increased rapidly and reached the peak value nearly at the same time as the peak runoff in the Red Soil region of southern China, then soil loss decreased and runoff leveled out^63^. Generally, the typicality of rainstorm erosion in loess hilly region are mainly manifested in several stages: First, because of differences of soil and topography, overland flow is often collected together from sheet overland flow and small rill flow, and causes rill erosion. Second, with the continuity of the rainfall-runoff process, the small rill flow also gradually increases, it will form shallow gully, dissected valley, gully and gravity erosion on steep slopes before entering into large ditches or streams. Third, infiltration-excess runoff on slopes pours into dissected valleys through overflow, rill flow and shallow gully flow, and then flows into river tributaries, again by different levels of ditches. Fourth, due to runoff erosion, gravity erosion and the increased sediment carrying capacity, the sediment carrying flow increases sediment concentration with the rising of the channel water-level. However, the increased amplitude of sediment concentration does not often correspond to the increase in river flow, and the occurrence time of the flood peak is not always consistent with the sand peak^31^.

Figure 10(b) indicates that the sand peak was always behind the flood peak, both on 14 July 2000 and 21 August 1998. Generally, runoff erosion can be fully developed by the long runoff convergence time, the sediment carrying capacity increases with increased river flow, and peak values of sediment concentration often appear at the same time or lag behind the flood peak. However, on the loess hilly and gully region, as soil water content increases in the late period of runoff yield, the shear strength of loess decreases, runoff erosion increases, and gravity erosion also frequently occurs, which makes the probability of the sand peak lagging behind the flood peak increase. The results are consistent with Dugan *et al*. (2009) who demonstrated the importance of antecedent moisture condition as an important factor in the rainfall runoff and sediment transport response to precipitation events^64^. The fundamental cause of erosion changes lies in rainfall characteristics, the soil erosion quantity caused by high rainfall intensity at a certain stage of an event-based rainstorm accounted for most soil erosion^65^.

**Figure 11:**
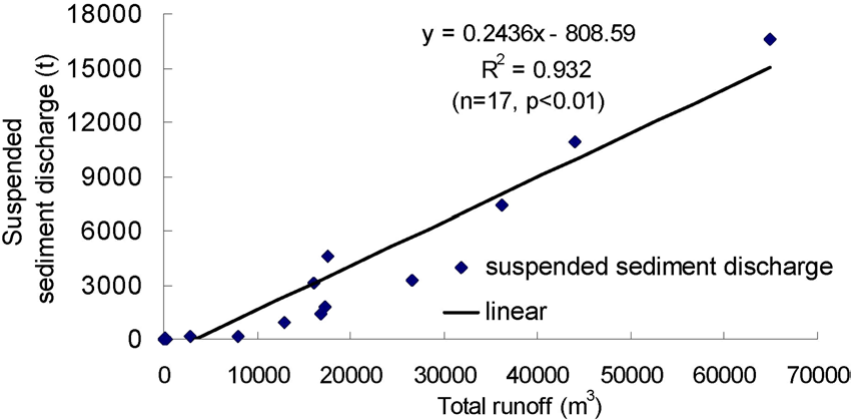
Regression analysis curve between total suspended sediment discharge and total runoff using 17 typical observed rainstorm events.

Figure 11 shows linear regression between total suspended sediment discharge and total runoff using 17 typical observed rainstorm events. In the flood rising section of flow hydrographs, the runoff-sediment data points often fall below the regression equation line, while in the flood falling section, the data points are above the regression line, indicating that sediment transport processes lag behind flow processes (Fig. 11). Such behaviour is consistent with land cover since, although sparse, the secondary forest and shrub land promotes shading and inhibits development of an herbaceous layer, favouring the sediment detachment process and decreasing surface roughness^66^. Among the three relationships of rainstorm, runoff and sediment yield, runoff amount has a strong correlation with sediment yield, and they can be statistically regressed as simple linear or approximate exponential associations. The results are consistent with Guo *et al*. (2017) that the relationship between runoff and sediment yield under the different land disturbances could be described by an exponential function^67^. Therefore, the corresponding association between sediment transport rate and flow processes can be quantitatively expressed by a mathematical model. Total sediment yield may be quantitatively estimated by the runoff-sediment relationship under different rainstorm events in the Zhifanggou Watershed. However, statistics show that the particularly large sediment concentration is often caused by the collapse of steep loess slopes, due to the flow scouring effects on the gully slope control line. But there is some uncertainty regarding the collapse of steep loess slopes, so the occurrence of large high sediment content also has some randomness. Besides, Poesen *et al*. (2003) found that collapse of stream banks and gully formation increased specific sediment yield, which occurs more intensively when soil is unprotected upon land use changes^68^. Both these phenomena will cause the scattered correlation between runoff and sediment concentration.

### Cluster Analysis of different event-based rainfall samples

**Figure 12:**
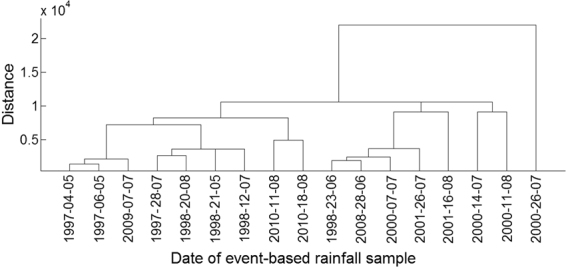
Cluster Analysis results of different event-based rainfall samples in Zhifanggou Watershed.

Soil conservation is the ultimate goal of sediment yield research^69^. Statistical techniques such as hierarchical cluster analysis and regression analysis with multiple grouping variables have been widely used to study soil erosion responses under different rainfall and runoff patterns^70^. Previous studies have classified regional rainfall events to evaluate the impact of rainfall characteristics on runoff generation, erosion and sediment processes in a semi-arid region based on rainfall depth, rainfall duration and 30-minute maximum rainfall intensity^71,72^. In this study, Cluster Analysis was used to assess individual rainstorm-erosion events similarities using 17 rainstorm-erosion event parameters. Figure 12 shows Cluster Analysis results for different event-based rainstorm samples in Zhifanggou Watershed. The three rainstorm events of 4 May 1997, 6 May 1997 and 7 July 2009 have similar characteristics, and they are classified into one category in Cluster Analysis. The runoff yield times of the three rainstorms are all between 83–150 minutes. The average flow is between 0.005–0.01 m^3^/s, peak flow is between 0.009–0.012 m^3^/s, and runoff depth is between 0.002–0.01 mm. Runoff coefficients were all small for these three rainstorms due to small rainfall intensity and runoff yield.

Cluster Analysis identified major differences between the rainfall-runoff erosion event on 26 July 2000 and other rainstorm events. The time of runoff yield in this rainstorm event was not the longest, but the peak flow, total flow and suspended sediment transport rates were all in the largest category. Flood duration is one of the main factors affecting the type of rainstorm erosion events in the basin. Specifically, the rainstorm duration was short during this rainstorm event, and rainfall intensity changed quickly. Rainfall intensity exceeded the soil infiltration rate, which makes rainstorms generate infiltration-excess runoff and form peak flow and then cause severe soil erosion. However, the two rainstorms (23 June 1998 and 28 June 2008) both occurred in June, they have similar rainfall duration, and the total runoff amounts were 16051.6 and 16774.8 m^3^, respectively. The difference of these indexes is small in the two rainstorm-erosion events, but there is a major difference for the modulus of sediment yield, they are 379.8 and 168.6 t/km^2^, respectively. Firstly, the large-scale returning farmland project was implemented on the Loess Plateau in 1997, by 2008 the preliminary governance results were achieved, so the total runoff amount may be large, but the sediment yield decreased due to vegetation interception. This indicates that the regulation of soil and water conservation measures on spatio-temporal scale effects of the rainstorm-erosion process is the key to control sediment output from the basin^73^. At the same time, the increase of vegetation coverage will increase rainfall interception^74^, reduce the kinetic energy of raindrops^75^, weaken rainfall erosivity^76^, and reduce runoff and sediment yield^77^. Secondly, the main reason for the sediment yield difference between 1998 and 2008 cannot be fully attributed to afforestation and grass planting. Because in this case the amount of rainfall, rainfall intensity and peak flow may play important roles in the large difference of sediment yield modulus. The high intensity and short duration of heavy rainstorms are the main factors accelerating storm runoff erosion in this watershed. Previous studies indicate that gully erosion cannot be effectively controlled by vegetation measures in the loess hilly areas with high valley density and deep dissected valleys^78^. Although vegetation cover significantly increased and soil erodibility significantly decreased with vegetation development and age of restoration, soil infiltration rates decreased. Thus the runoff coefficient and the runoff volume both increased, then the watershed gully erosion and sediment yield also increased^27^. Similarly, Xu *et al*. (2017) and Wu *et al*. (2017) both found that soil loss increased with increased rainfall intensity^79,80^.

A small watershed is both the main source of regional sediment yield and the basic unit of erosion control and ecological environment restoration^81^. Table 1 shows runoff and sediment parameters and cluster results of different event-based rainstorms in Zhifanggou Watershed. The cluster results indicate that 17 rainstorms can be broadly divided into two major categories: (i) The first is the strongest rainstorm event, which reflects the maximum peak discharge, and the maximum suspended sediment discharge, such as the rainstorm that occurred on 26 July 2000. The average flow rate in this rainstorm was 8.9263 m^3^/s, the peak flow rate was 17.2954 m^3^/s and the total flow was 16774.84 m^3^. This shows that the great rainstorm produced large runoff and very intensive erosion, and the concentrated runoff is a dynamic condition for strong erosion of loess soil. (ii) The second is the rainstorm event which reflects the individual peak discharge and suspended sediment transport, which forms the other 16 rainstorm events. The second category can be subdivided into two smaller categories: one category is the rainstorm event with average flow >1.3 m^3^/s and suspended sediment amount >1300 t; the other category is the rainstorm event where the suspended sediments are <1000 t. The two small categories can also be subdivided into many small categories at the next level. Therefore, Cluster Analysis can be used to measure the similarity of different rainfall erosion events. Based on regression and Cluster Analyses in Figs 3–9 and Table 1, the criterion of relationships between sediment yield and runoff under typical rainstorm events in Zhifanggou Watershed can be statistically summarized as follows. For rainstorm events of sediment modulus >1000 t/km^2^, the power function of the sediment-runoff fitting effect is better than the logarithmic function. For rainstorm events of sediment modulus between 300–1000 t/km^2^, the logarithmic function of the sediment-runoff fitting effect is superior to the power function. For rainstorm events of sediment modulus <300 t/km^2^, linear associations are stronger. Therefore, classifying and describing the similar characteristics of runoff erosion in different rainstorm events can both provide important data support for soil conservation planning in a watershed, and provide a strong theoretical basis for establishing soil erosion prediction models. So, Cluster Analysis is a useful tool to evaluate characteristics of different rainstorm erosion events and to understand soil erosion and sediment transport processes.

## Conclusions

Taking Zhifanggou Watershed of 8.27 km^2^ in the loess hilly and gully region as the study area, the characteristics of runoff erosion and sediment yield based on 17 observed rainstorm events from 1997–2010 were statistically evaluated. The pulsed rainstorm is both the original driving force of slope soil erosion, and one of the most important factors influencing sediment transport processes. The main power of water erosion is rainfall and surface runoff, the amounts of runoff and erosion are closely related to rainfall intensity and rainfall amount. However, correlations of rainfall-runoff and rainfall-sediment during different rainstorms are often scattered and the correlation coefficients are often weak, due to effects of the infiltration-excess runoff and soil conservation measures in the loess hilly region. Runoff has a strong correlation with sediment yield. It can be expressed by simple exponential or linear equations. The sediment transport modulus can be quantitatively estimated by the corresponding runoff-sediment correlation under different rainstorm events. The response characteristic of sediment yield simulation is variable in different levels of pulsed runoff-erosion events. Affected by hyper-concentration flows, great changes have occurred in the interactions between flow and sediment concentration for different flood events. For rainstorm events of sediment modulus >1000 t/km^2^, the power function of the sediment-runoff fitting effect is stronger than the logarithmic function. For rainstorm events of sediment modulus between 300–1000 t/km^2^, the logarithmic function of the sediment-runoff fitting effect is superior to the power function. For rainstorm events of sediment modulus <300 t/km^2^, linear relationships are stronger. This new overall understanding opens opportunities for decision-makers and managers to conserve soil based on observation data of rainstorm erosion in small watersheds.
